# The global hepatitis delta virus (HDV) epidemic: what gaps to address in order to mount a public health response?

**DOI:** 10.1186/s13690-021-00693-2

**Published:** 2021-10-19

**Authors:** Tomoyuki Hayashi, Yumie Takeshita, Yvan J.-F. Hutin, Hande Harmanci, Philippa Easterbrook, Sarah Hess, Judith van Holten, Ena Oghenekaro Oru, Shuichi Kaneko, Cihan Yurdaydin, Marc Bulterys

**Affiliations:** 1 Global Hepatitis Programme, World Health Organization, Geneva, Switzerland; 2 Department of Gastroenterology, Kanazawa University and WHO Collaborating Center for Chronic Hepatitis and Liver Cancer, Kanazawa, Ishikawa Japan; 3 Department of Gastroenterology, Ankara University School of Medicine, Ankara, Turkey; 4 Hepatology Institute, University of Ankara, Ankara, Turkey

**Keywords:** Hepatitis delta, HDV, Hepatitis B virus, Prevalence, Superinfection, Novel therapeutic strategies, Cirrhosis, Hepatocellular carcinoma

## Abstract

**Background:**

Co-infection between hepatitis B virus (HBV) and hepatitis delta virus (HDV) causes the severest chronic hepatitis and is associated with a high risk of cirrhosis and hepatocellular carcinoma (HCC). The Global Health Sector Strategy on Viral Hepatitis called for the elimination of hepatitis (− 65% mortality and − 90% incidence) by 2030. Our aims were to summarize key points of knowledge and to identify the gaps that need to be addressed to mount a public health response to HDV.

**Methods:**

We performed a current literature review in terms of epidemiology by WHO regions, genotypes distribution and their pathogenicity, factors associated with HDV infection, mortality due to HDV infection, testing strategies and treatment.

**Results:**

Prevalence of infection and genotypes are heterogeneous distributed, with highest prevalence in foci around the Mediterranean, in the Middle East, and in Central, Northern Asia and Eastern Asia. Persons who inject drugs (PWID) and migrants from highly endemic areas are highly affected. While antibody detection tests are available, HDV RNA tests of current infection are not standardized nor widely available. The few therapeutic options, including lofartinib, are not widely available; however several new and promising agents have entered clinical trials.

**Conclusion:**

HDV infection is an poorly known cause of chronic liver disease. To mount a public health response, we need a better description of the HDV epidemic, standardized testing strategies and better treatment options.

## Background

Of the viruses causing hepatitis, Hepatitis delta Virus (HDV) is unique in that it needs the helper function of Hepatitis B Virus (HBV) to infect hepatocytes [1]. The World Health Organization (WHO) estimates that in 2015, 257 million people (3.5% of the world’s population) were infected with HBV [2]. However, WHO does not have estimates of prevalence or mortality for HDV. Co-infection of an HBV infected person with HDV worsens the outcome, with higher rates of cirrhosis and hepatocellular carcinoma. Most published reviews quote 5% as an estimate of the prevalence of HDV coinfection among persons with HBV infection (about 13 million persons worldwide) [3], mostly in high endemicity foci or among immigrants from highly endemic regions [4].

In 2016, the Global Health Sector Strategy (GHSS) on Viral Hepatitis [5] called for the elimination of hepatitis (− 65% mortality and − 90% incidence) by 2030. The prevention components of the GHSS (e.g., HBV immunization, blood and injection safety programmes) will prevent HBV infection and thus HDV infection. However, the GHSS provides limited solutions for HDV infection from a testing and treatment perspective. In recent two meta-analyses, the global prevalence had been estimated to be 0.8–0.98% in the general population, and 13–14% in the HBsAg-positive population, which corresponds to around 60 million infections globally [6, 7].

A number of professional associations [8–12] address hepatitis D in their care and treatment guidelines (Table 1). Several reviews summarized the landscape in epidemiology [13, 14], testing [15], or treatment [16]. However, these did not address HDV care from a public health perspective. We therefore performed a current literature review in terms of epidemiology by WHO regions, genotypes distribution and their pathogenicity, factors associated with HDV infection, mortality due to HDV infection, testing strategies and treatment. Our aims were to summarize key points of knowledge and to identify the gaps that need to be addressed to mount a public health response to HDV.

## Epidemiology by WHO regions

**Fig. 1:**
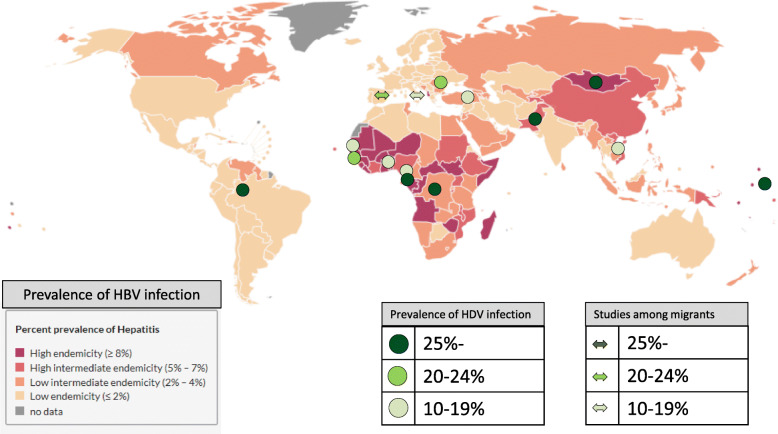
Geographical location of studies reporting high prevalence of HDV infection among HBV infected people, in all ages, worldwide, 2008–2017

Recent review estimated 12 million people worldwide have experienced HDV infection. The geographic distribution of HDV infection is heterogeneous [17]. Among HBV infected persons, HDV infection is particularly common in Central and West Africa, Central and Northern Asia, Viet Nam, Mongolia, Pakistan, Japan, the Taiwan province of China, Pacific Islands (Kiribati, Nauru), the Middle East, Eastern Europe (e.g., Turkey), South America (the Amazone basin), and Greenland [8, 18] (Fig. 1).

### Africa

In a 2017 systematic review, HDV antibody (anti-HDV) prevalence among HBV-infected persons varied from 26% in Central Africa, 7% in West Africa, to only 0.05% in Eastern and Southern Africa. High-prevalence spots were reported in Gabon (45%, 2015), Democratic Republic of the Congo (26%, 2017), Mauritania (19%, 2009), Cameroon (14–35%, 2011) and Nigeria (5%, 2014) in the general HBV infected population. Among HIV-HBV co-infected persons, high prevalence spots were reported in Guinea-Bissau (25%, 2011), Cameroon (12%, 2010) and Nigeria (7%, 2004) [14].

### The Americas

HBV mostly affects Indigenous Populations in the Americas. In the Amazon region of North Brazil, in 2003–2009, anti-HDV prevalence was 29%. among HBV infected persons [19, 20]. Anti-HDV prevalence in indigenous populations with HBV infection was 39% of Peru, 13% in Brazil, 8% in Colombia, and 4–6% in Venezuela [21]. In the United States, anti-HDV prevalence among HBV-infected persons was around 3% [22] between 2012 and 2016. Advanced fibrosis was associated with high HDV viral load in the western Amazon Basin of Brazil [23]. From 2008 to 2014, age-standardized mortality rates due to HDV were much higher in the North region of 2.2 per million compared to all regions of 0.28 per million [19].

### Eastern Mediterranean

A 2010, a systematic review estimated that anti-HDV prevalence was 15% in persons with HBV infection (pooled prevalence: 25% in Sudan, 18% in Pakistan, 16% in Tunisia, 11% in Egypt, 7% in Saudi Arabia, 5% in Iran, and 2% in Yemen) [13]. In Pakistan, there is a high prevalence belt in the rural Sindh province [24]. In other countries, one study was available that reported 29% in Afghanistan, 17% in Somalia, 2% in Jordan, 2% in Djibouti, and 0.9% in Lebanon [14].

### Europe

In Europe, areas reporting high prevalence of anti-HVD among HBV infected persons include Romania (23%, 2015) [25], Eastern Turkey (15%, 2012–2014 [26], higher than in western Turkey (3%) [27], Yakutia, Siberia, Russia (18–20%, 1996–1998) [28], and Greenland, among Inuits [29, 30] (6%, 2011) [31]. In Austria, Belgium, Bulgaria, Czech Republic, Croatia, France, Greece, Ireland, Poland, and Switzerland, HDV is uncommon [32].

### South-East Asia

In Bangladesh, 22% of HBV infected persons were anti-HDV positive in 2003 [33]. In New Delhi, India, a 11% anti-HBV prevalence was reported in HBV infected persons in 2005 [34]. In others areas with high prevalence of HBV such as Indonesia [35] and Thailand [36], HDV is uncommon [24].

### Western Pacific

In Mongolia, where hepatocellular carcinoma (HCC) is the most common cancer (annual rate: 54.1 cases per 100,000 people) [37], anti-HDV prevalence among HBV infected perons was 45% in 2013 [37] (60% in 2017 when using a novel quantitative microarray antibody capture assay) [38]. In China, 4% of persons with HBV infection were anti-HDV positive in Inner Mongolia, Xinjiang and Tibet, where ethnic minorities live [39]. The Taiwan province of China was endemic for HBV, but following immunization, the prevalence of HBV and HDV infection decreased. However, anti-HDV was more common in the south than in the north (6% vs 2%) [40]. In Japan, HDV is highly prevalent in the Miyako Islands (24% in one of these islands in 1997 [41]) [42]. The distribution of anti-HDV in the Pacific is highly heterogeneous, with reports in Nauru, Kiribati [43], Niue and Western Samoa while It is extremely low or absent in other Islands [44, 45]. In Viet Nam, in 2000–2009, 15% of HBsAg positive people were HDV-RNA positive [46]. In Australia, Malaysia, Philippines and the Republic of Korea, HDV is uncommon in the general HBV infected population [39].

## Genotypes distribution and their pathogenicity

There are at least eight HDV genotypes (1 to 8). Viral genotypic diversity is related to the geographical location (Table 2). HDV genotypes differ in terms of clinical outcomes. Genotype 1 is prevalent worldwide and has a variable course of infection. In Europe, North America and South Asia,Central and Northern Asia, eastern Mediterranian, and Middle East, genotype 1 is the most prevalent [47–57], while others are extremely rare. Genotype 1 was the most prevalent in Africa (median 88%) [14]. Genotype 2 is mostly reported from the Yakutia region of Russia, the Taiwan province of China, and Japan [28]. It possibly derives from a common genotype 2 prototype [28]. Genotype 2 is associated with higher rate of remission than genotype 1 [58]. Genotype 3 is common in the Amazon Basin (90% of HDV-infected people) [20], and is associated with more severe forms of the disease, earlier onset of HCC and outbreaks of fatal acute liver failure [59]. Genotype 4 is reported in the Far East and often leads to mild liver disease. However, a variant of genotype 4 on the Miyako Islands in Japan is associated with greater progression to cirrhosis than genotype 4 in the Taiwan province ofChina [60]. Genotypes 5–8 are reported in Africa and in African migrants to Europe [14, 61, 62], but the natural history is not well characterised [14, 63, 64].

**Table 1:** Key elements regarding hepatitis D in the guidelines addressing hepatitis B

	Epidemiology	Natural history	Screening, Diagnosis	Treatment	Management	Prognosis
AASLD	–	–	Anti-HDV screening is recommended in HIV positive persons, persons who inject drugs, men who have sex with men, those at risk for sexually transmitted diseases, migrants from areas of high HDV endemicity, patients with low HBV-DNA levels and elevated ALT levels.	PegIFNα for 12 months is the recommended therapy for those with elevated HDV-RNA levels and ALT elevation. If HBV-DNA levels are elevated, concurrent therapy with NA is indicated.	Assessment of HDV-RNA is warranted if ALT elevation occurs following treatment because of the high rates of relapse. Reasonable to refer patients to specialized centers that offer access to experimental therapies.	–
EASL	–	Severe or fulminant hepatitis is more frequently reported in HBV-HDV co-infection compared to HBV mono-infection. Chronic infection after acute HBV-HDV hepatitis is less common, while chronic delta hepatitis develops in 70–90% of patients with HDV superinfection.	Confirmed by detectable HDV RNA, immuno-histochemical staining for HDV antigen, or IgM anti-HDV. However, diagnosis of active HDV infection may be difficult, as HDV RNA assays are not standardised and HDV antigen and IgM anti-HDV assays are not widely available.	PegIFNα for at least 48 weeks is the current treatment of choice in HDV-HBV co-infected patients with compensated liver disease. It can be continued irrespective of on-treatment response pattern if well tolerated. Patients with ongoing HBV DNA replication, NA therapy should be considered.	Long-term follow-up HDV RNA monitoring is recommended for all treated patients as long as HBsAg is present.	Persistent HDV replication leads to cirrhosis and HCC at annual rates of 4 and 2.8%, leading to high fatality rate and justifying the need for antiviral therapy.
APASL	The prevalence of HDV has not declined. In the United States of America, Australia and some European countries, the prevalence of HDV infection is increasing.	Chronic infection after acute HBVHDV hepatitis is less common, while chronic hepatitis D develops in 70–90% of patients with HDV superinfection.	Confirmed by detectable HDV RNA, immuno-histochemical staining for HDV antigen, or IgM anti-HDV. But diagnosis of active HDV infection may be difficult, as HDV RNA assays are not standardized and HDV antigen and IgM anti-HDV assays are not widely available.	Pegylated interferon is effective against HDV. Weekly injection of pegylated interferon is currently used for 12–18 months. Nucleotide analogues treatment might be considered in some patients who have active HBV replication with persistent or fluctuating serum HBV DNA levels above 2000 IU/ml.	Patients should be monitored for 6 months post treatment and beyond.	HDV can cause severe liver injury that may result in fulminant hepatic failure and rapid progression to cirrhosis and hepatic decompensation, as well as an increased risk of liver cancer.
WGO	Up to 5% of the world’s population is infected with HBV, and probably 5% of those chronically infected with HBV have HDV infection. Some endemic areas in the developing world may have much higher prevalence.	Coinfection evolves to chronicity in only 2% of cases, but is associated with a higher chance of fulminant acute infection, while superinfection leads to progressive disease and cirrhosis in more than 80% of cases.	Should be evaluated particularly if hepatitis is present in the face of little or no HBV viral replication, or if they come from an HDV-endemic region or have acquired HBV through injection drug use. Infection should be diagnosed by detection of HDV RNA in serum by polymerase chain reaction, or indirectly by detection of antibodies against hepatitis D antigen of the IgG and IgM classes.	Chronic hepatitis D should be treated with IFN (preferably pegyIFN) for at least 12 months, but the treatment results are suboptimal. Patients with active HBV replication despite HDV coinfection may benefit from treatment with NA in combination with PegIFN.	–	Cirrhosis develops at a younger age than in patients with chronic HBV monoinfection.

AASLD, American Association for the Study of Liver disease; EASL, European Association for the Study of the Liver; APASL, Asian Pacific Association for the Study of the Liver; WGO, World gastroenterology Organisation Global Guideline

NA, nucleos(t) ide analogue; IFN, interferon; PegIFN, pegylated interferon

**Table 2:** HDV genotypes and their geographical distribution

Genotype	Geographical location	Clinical features
1	Europe, North America and South Asia,Central and Northern Asia, eastern Mediterranian, and Middle East	Variable course
2	Yakutia region (Russia), Taiwan (China), and Japan	Higher rate of remission than genotype 1
3	Amazon Basin	More severe, earlier onset of HCC, and outbreaks of lethal acute liver failure
4	Miyako Island (Japan), Taiwan (China)	Variant on the Miyako Islands associated with greater progression to cirrhosis than in Taiwan
5	Africa, Europe (migrants from Africa)	More associated with HCC than genotype 1 and other African genotypes Better prognosis with fewer episodes of hepatic decompensation
6,7,8	Africa, Europe (migrants from Africa)	Few data

## Factors associated with HDV infection

An association between prevalent HDV infection and injection drug use was reported the USA [65, 66], United Kingdom [67], and Iran [9, 12]. The high prevalence of anti-HDV in PWIDs suggests that injecting drug use is a risk factor for HDV super-infection. Sex workers also often have a higher prevalence of anti-HDV, especially if they inject drugs [68]. Migration from high HDV infection prevalence countries affects the epidemiology of the host country, explaining an increasing prevalence in France [69], Greece [70], Italy [71], Spain [72], Germany [73], and the United Kingdom [67]. In Western European countries, 55 to 95% of HDV infections are reported in more affected population groups, including PWID, and immigrants from highly endemic areas [16].

## Mortality due to HDV infection

The International Agency for Research on Cancer estimated that in 2012, around 430,000 (56%) of new HCC cases were attributable to HBV [74, 75]. WHO reported that in 2015, approximately 380,000 people died of liver cancer secondary to HBV and 490,000 people died of cirrhosis due to HBV [76]. While the HDV coinfection worsens the outcomes of patients infected with HBV, very few studies estimated the proportion of HBV deaths that are also attributable to HDV infection.

## Testing strategies

Among persons with HBV infection, clinical guidelines recommend testing for HDV infection in patients who migrated from high prevalence areas, in those with cirrhosis, and those who have elevated liver tests while receiving appropriate treatment for HBV infection [8, 12]. In highly endemic areas, HBsAg positive patients may need to be tested at least once for anti-HDV. In other areas, testing may be restricted to HBsAg positive persons with abnormal ALT and low HBV DNA and those not responding well to appropriate treatment, irrespective of HBV DNA level. In terms of cancer prevention, some clinical guidelines recommend screening persons with HDV infection with ultrasonography every 6 months [8].

## Treatment

The management of HDV infection has not changed in over 30 years and consists of treatment with interferon. However, new medicines have been explored for the management of HDV [16]. Furthermore, novel therapeutics that target HDV entry, prenylation and nucleic acid replication now offer a promise of more effective treatment for HDV infection [77].

### Standard therapies

Pegylated interferon (PegIFN) is the only medicine mentioned as effective against HDV in the WHO guidelines [78]. Other guidelines recommend PegIFN for at least 48 weeks irrespective of response patterns [8, 10–12, 79]. Of patients who receive PegIFNα for 48 weeks, 17–43% of treated patients had undetectable HDV RNA [80–83]. Sustained virological response remains uncommon, and late relapses occurred in more than 50% of responders [84]. Stopping PegIFN prematurely is not recommended if treatment is well tolerated. Late responses may occur in patients and long-term follow-up studies indicate that IFN based therapy is associated with a lower disease progression. Long-term follow-up and HDV RNA monitoring is recommended for all treated patients as long as HBsAg is present.

### Nucleotide analogues

WHO recommends Nucleot(s) ide analogues (NA) for the treatment of chronic HBV infecton [2, 6]. NA do not impact HDV replication and related disease, because the virus uses host enzymes for replication and thus lacks enzyme targets. NA analogs have been tested for a duration of 6 to 18 months in chronic hepatitis D and were ineffective (NAs do not affect HBsAg synthesis, the main function needed by HDV for propagation) [85]. Tenofovir was reported to have an effect when used for longer treatment duration in HIV-HDV co-infection [86, 87], although these results have not been confirmed [88, 89].

### Nucleotide analogs in combination with interferons

A trial compared 48 weeks pegylated interferon with or without adefovir with adefovir monotherapy 28% of patients receiving pegylated interferon with or without adefovir (compared to none on adefovir) achieved undetectable HDV-RNA level 24 weeks after completing therapy [90]. A similar effect on HBsAg levels was not reported in a follow up study, which compared PegIFN-tenofovir combination with PegIFN monotherapy [91]. Adding lamivudine [92, 93], ribavirin [83, 94], adefovir [90], tenofovir [88], or entecavir [95] to PegIFN does not provide additional benefits over monotherapy.

### Myrcludex B

Myrcludex B is a hepatocyte entry inhibitor that interferes with HDV entry [96, 97]. It reduces the population of HDV-positive cells and allows HDV-free hepatocytes to regenerate, which might ultimately lead to eradication of the virus [98]. In a phase 2a study of patients HBV-DNA and HDV-RNA positive, myrcludex B led to HDV RNA decline, and 1 of 7 patients became HDV-RNA negative. Moreover, 5 of 7 patients became HDV-RNA negative in combination with PegIFN, suggesting a synergistic effect of myrcludex B and PegIFN [99]. In a phase 2b open label study, Myrcludex B in combination with tenofovir led to a dose-dependent HDV-RNA decline associated with clinical [100] and virological [101] improvements. However, HDV-RNA levels rebounded after Myrcludex B was discontinued [102]. Myrcludex B has been well tolerated with no dose limiting toxicity [101]. In a new phase 2 study suggested a synergistic effect of myrcludex B in combination with pegylated interferon [103]. However, asymptomatic dose-dependent increases in bile acid levels were reported in study participants.

### Lonafarnib

Lonafarnib is a prenylation inhibitor that inhibits virion assembly. In a phase 2 double-blinded, randomized, placebo-controlled trial, oral lonafarnib (100 mg or 200 mg) twice daily for 28 days significantly reduced viremnia [104]. However, serum HBsAg levels did not change and adverse events were frequent [101]. Combining lonafanib with PegIFN achieved more substantial and rapid HDV-RNA reduction, compared to historical responses with PegIFN alone.

### Ritonavir

Ritonavir (a protease inhibitor) inhibits one of the cytochrome P^− 450^ systems that metabolizes lonafarnib, leading to higher serum levels with minimal side effects [104, 105]. Lonafarnib plus ritonavir yielded a better antiviral response RNA after 8 weeks of therapy [102], which did not last after the therapy was discontinued [106]. However, in a subset of patients, 8 to 12 weeks of therapy with a lonafarnib based regimen, led to a post-treatment biochemical flare with subsequent normalization of ALT and undetectable viruses [104]. Tiple therapy with low dose (either 25 mg, bid or 50 mg, bid) in combination with ritonavir for 24 weeks appears synergistic [107].

### Nucleic acid polymers

Nucleic acid polymers (NAPs) can lead to dramatic declines in HBsAg for HBV-HDV co-infected patients after 6 months or less. REP2139 is one of these NAPs and an inhibitor of HBsAg release. A phase 2 proof of concept trial combined REP2139 with PegIFN. Nine among 12 patients were negative at the end of treatment, 7 were negative at the end of the one-year follow-up, and 5 maintained HBsAg-suppression at the end of follow-up. Functional remission is stable at 1.5 years follow-up and is associated with persistently normal liver function and progressive reduction in median hepatic stiffness [108]. However, hematological adverse events were reported. On-treatment ALT flares may be a concern [109].

### Ezetimibe

Ezetimibe inhibits NTCP and therapy with ezetimibe may lead to a decline in HDV levels. A clinical phase 2 trial is recruiting (NCT03099278).

## Discussion: next steps from a public health perspective

### Epidemiology

There is extremely high variation in HDV prevalence and genotype distribution worldwide. Prevalence is declining because of HBV immunization, but epidemiological data in many countries remain scant. Risk behaviours associated with HDV infection are also unclear. Consequently, the global HDV prevalence has been difficult to describe precisely so far and more information is necessary. From a mortality perspective, if we assume that the prevalence of HDV infection is 5–10% in the 390,000 persons who died from HBV associated HCC and in the 480,000 persons associated cirrhosis in 2015 [2], then at least 19,500–39,000 cases of HCC deaths and 24,000–48,000 cases of cirrhosis deaths could be HDV related [76, 110].

### Testing

HDV prevalence is higher in PWID, sex workers, and migrants from highly HDV endemic countries. Thus, clinical guidelines [5, 9] often recommend testing in such priority populations and in high endemicity countries. However, testing approaches from a public health perspective require data on national and global epidemiology and evidence to guide how to test. While detection of anti-HDV-IgG is commonly used to test for HDV infection, HDV-RNA testing can be used to confirm infection. However, it is not widely available, and not yet standardized. Innovative methods such as point-of-care rapid diagnostic tests are not yet available. In addition, fibrosis staging of liver disease in HDV is challenging as non-invasive tests are impracticable [111]. Effective fibrosis score specifically developed for chronic HDV infection is necessary.

### Treatment

Therapeutic options for HDV infection are still limited and PegIFN and NA are the only medicines mentioned in the most recent hepatology society guidelines [8, 10–12]. However, none of these NAs affect HDV RNA replication per se. IFNs are of limited effectiveness and are associated with a high rate of relapse. New treatment strategies targeting different steps of the HDV life cycle are emerging and provide hope for the future for HDV co-infected patients. Some medicines appear more effective in combination with others, especially combined with PegIFN. Clinical trials are still ongoing and limited evidence is available. Sustained HDV suppression or cure would be necessary for sustained clinical improvement. However, since new treatments of HDV are an urgent need, experts on HDV treatment have recommended that a 2 log decline of HDV RNA at end of treatment from baseline should represent a surrogate for initial treatment efficacy in clinical trials of novel therapies for patients with CHD [112].

## Limitations

Our review suffers from a number of limitations. First, HDV RNA and genotype testing are not routinely done in most countries, therefore, relevant data are very limited. We therefore defined HDV infection as positive for IgG anti-HDV. Second, sampling often included potentially biased populations, such as medical outpatients or health-care workers. Finally, we summarized epidemiological data but there were insufficient data in the literature to analyse the long-term outcome of HDV infection. Long-term, multicenter prospective studies are needed to better understand the prognosis of HDV infection in different settings.

## Conclusion

Our review supports three conclusions. First, while certain countries and risk/ethnic groups have very high HDV prevalence, there is a lot of heterogeneity and many data gaps. As a result, reliable global HDV prevalence estimates are unavailable. Second, testing approaches have not been defined for HDV because of a lack of diagnostic tests and insufficient evidence to determine who needs to be tested. Third, few treatment options are currently available for HDV, but none are sufficiently effective, cost effective or affordable to allow delivery on a large scale. There are three areas of work that need to be undertaken to address these gaps. First, the regional and global epidemiology need to be better characterised using systematic reviews and meta-analyses, with generation of more surveillance data at the country level. Second, we need to develop a public health testing approach for HDV, that would address who to test and how to test. Third, researchers, clinicians, academia, funding/donor institutions and governments will need to work closely with each other to make sure that treatment options identified as promising can be further evaluated and scaled for access to those infected with HDV in line with the global goal of hepatitis elimination by 2030.

## Data Availability

All data generated or analysed during this study are included in this published article.

## Abbreviations

Anti-HDV: Hepatitis delta virus antibody
HBV: Hepatitis B virus
HDV: Hepatitis delta virus
HCC: Hepatocellular carcinoma
PWID: Persons who inject drugs
WHO: World Health Organization
GHS: Global Health Sector Strategy
PegIFN: Pegylated interferon
NA: Nucleot(s) ide analogues
